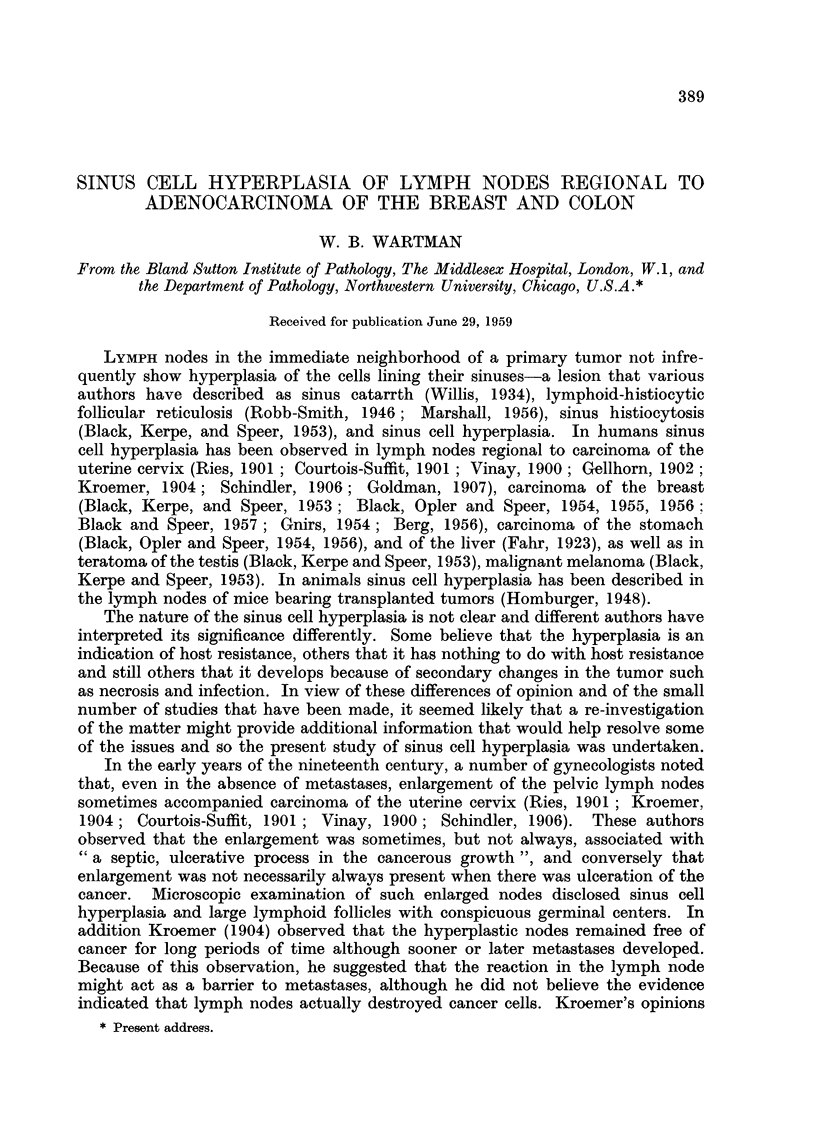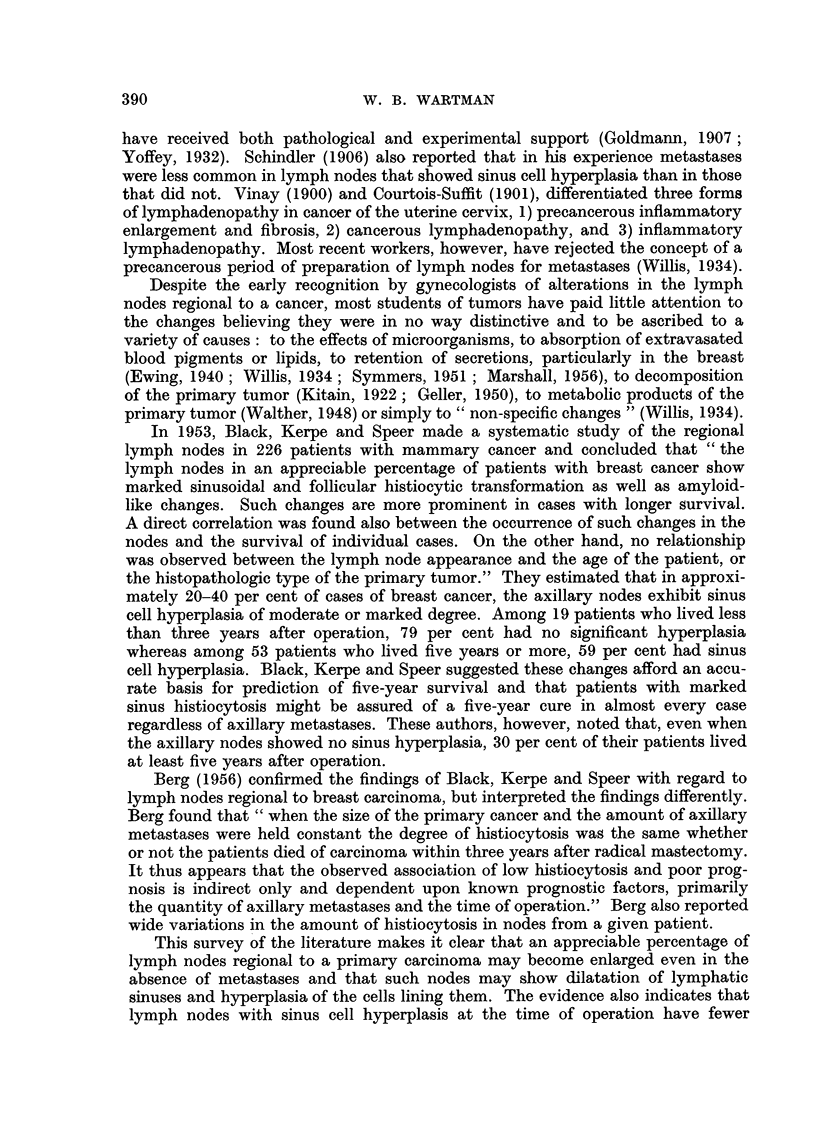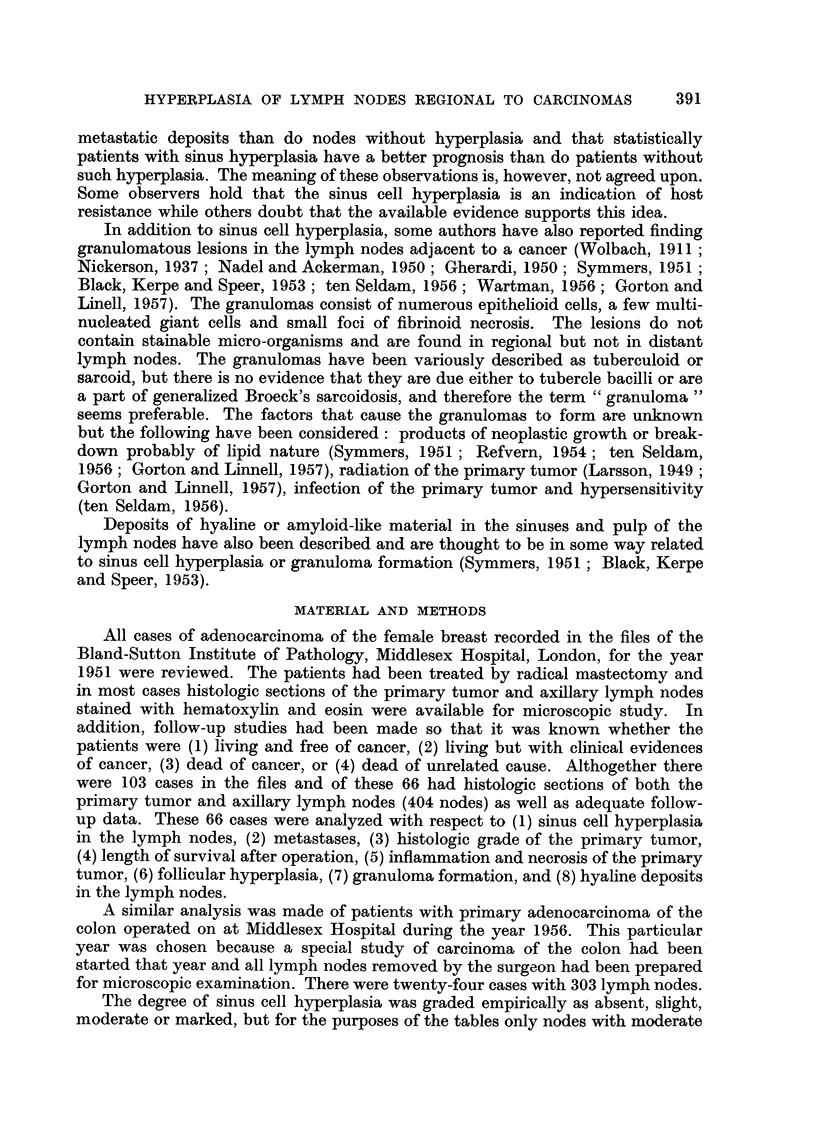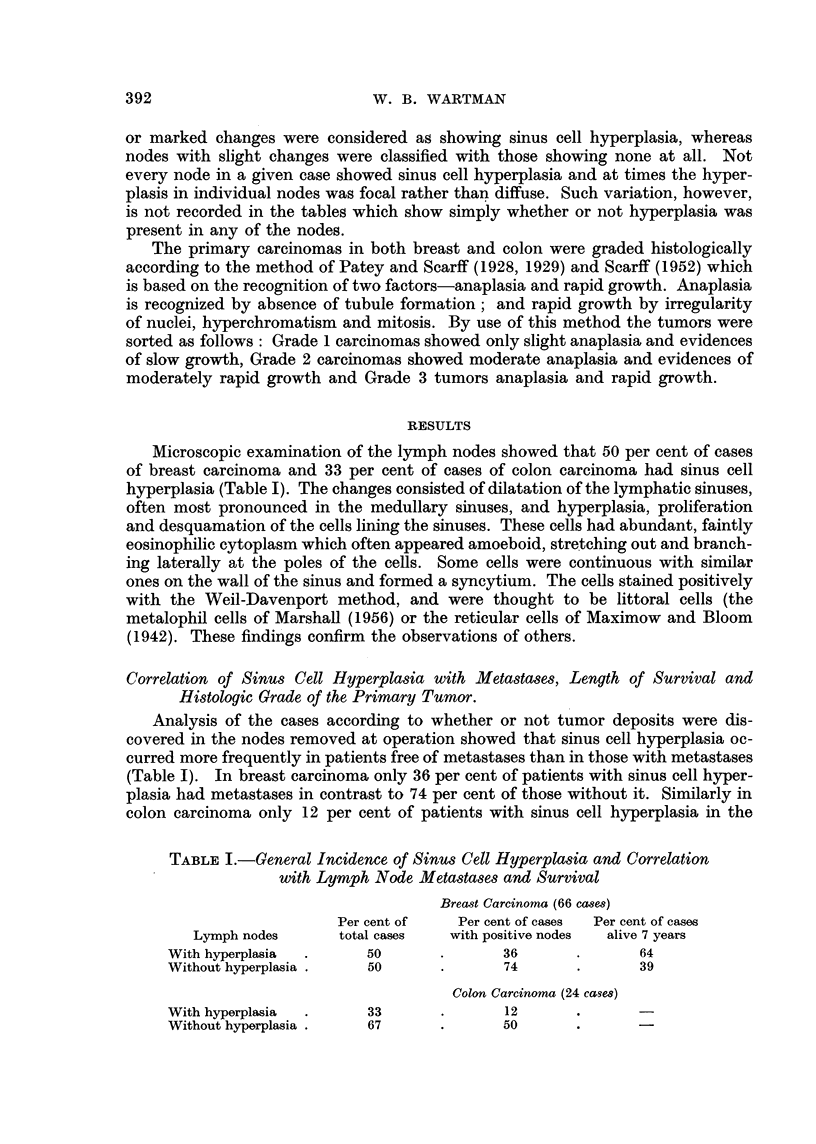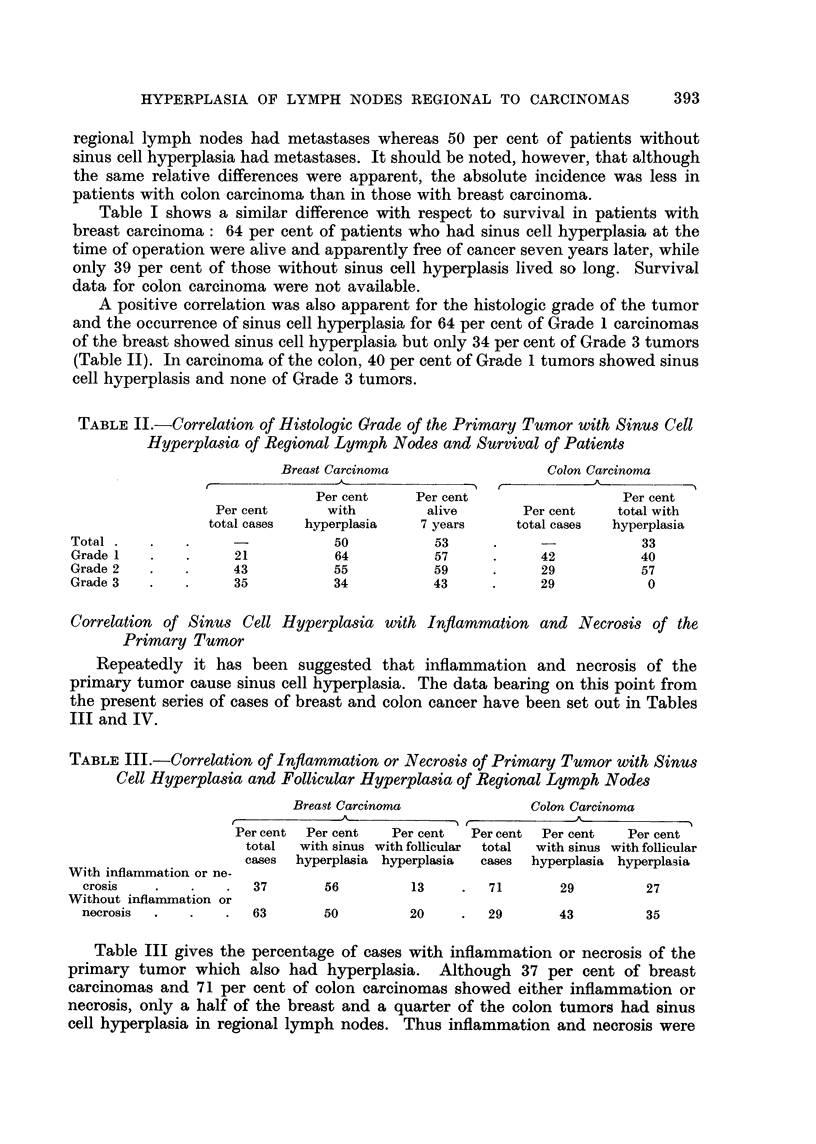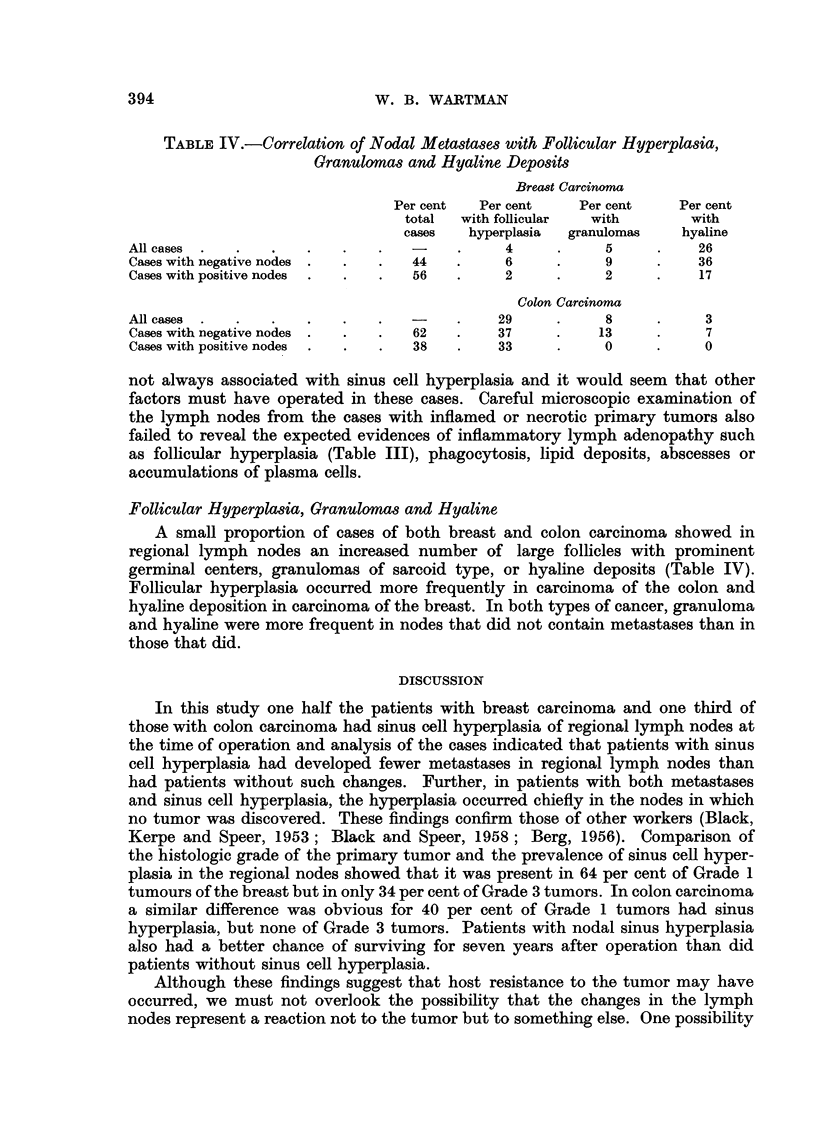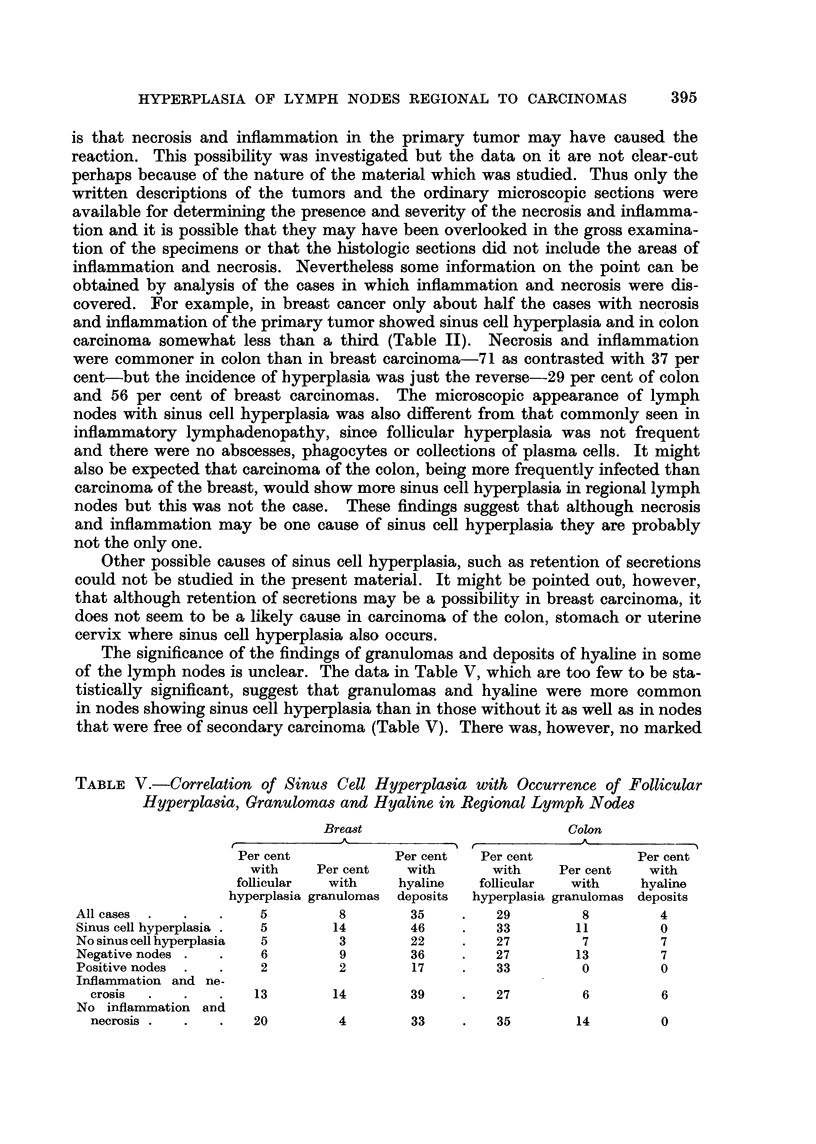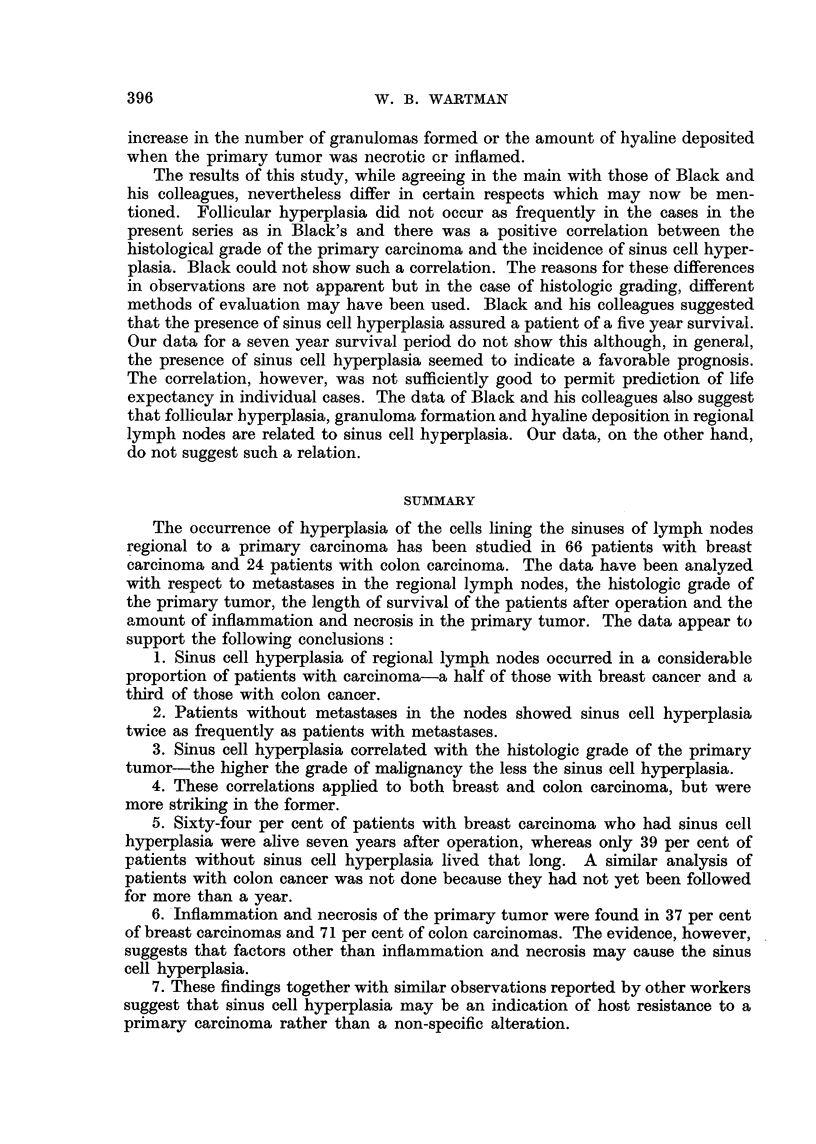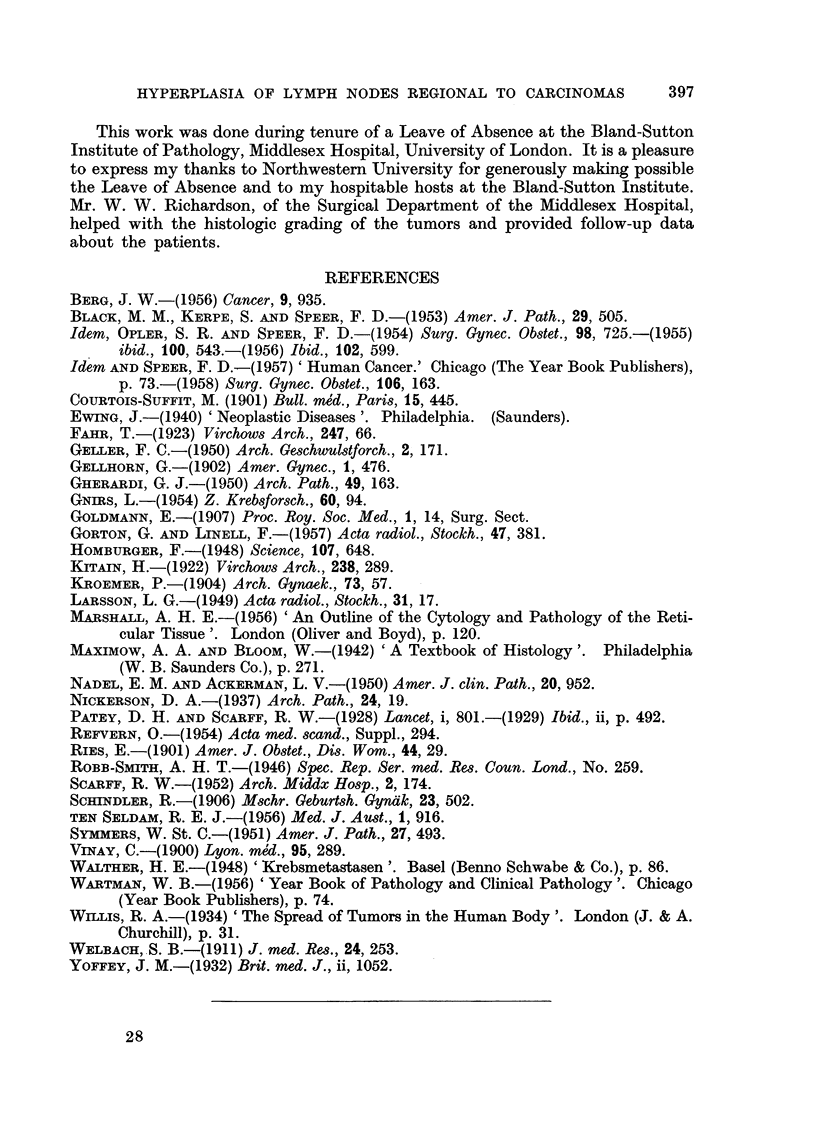# Sinus Cell Hyperplasia of Lymph Nodes Regional to Adenocarcinoma of the Breast and Colon

**DOI:** 10.1038/bjc.1959.44

**Published:** 1959-09

**Authors:** W. B. Wartman


### SINUS CELL HYPERPLASIA OF LYMPH NODES REGIONAL TO ADENOCARCINOMA OF THE BREAST AND COLON

W. B. WARTMAN

*From the Bland Sutton Institute of Pathology, The Middlesex Hospital, London, W.1, and the Department of Pathology, Northwestern University, Chicago, U.S.A.**

Received for publication June 29, 1959

LYMPH nodes in the immediate neighborhood of a primary tumor not infrequently show hyperplasia of the cells lining their sinuses—a lesion that various authors have described as sinus catarrth (Willis, 1934), lymphoid-histiocytic follicular reticulosis (Robb-Smith, 1946; Marshall, 1956), sinus histiocytosis (Black, Kerpe, and Speer, 1953), and sinus cell hyperplasia. In humans sinus cell hyperplasia has been observed in lymph nodes regional to carcinoma of the uterine cervix (Ries, 1901; Courtois-Suffit, 1901; Vinay, 1900; Gellhorn, 1902; Kroemer, 1904; Schindler, 1906; Goldman, 1907), carcinoma of the breast (Black, Kerpe, and Speer, 1953; Black, Opler and Speer, 1954, 1955, 1956; Black and Speer, 1957; Gnirs, 1954; Berg, 1956), carcinoma of the stomach (Black, Opler and Speer, 1954, 1956), and of the liver (Fahr, 1923), as well as in teratoma of the testis (Black, Kerpe and Speer, 1953), malignant melanoma (Black, Kerpe and Speer, 1953). In animals sinus cell hyperplasia has been described in the lymph nodes of mice bearing transplanted tumors (Homburger, 1948).

The nature of the sinus cell hyperplasia is not clear and different authors have interpreted its significance differently. Some believe that the hyperplasia is an indication of host resistance, others that it has nothing to do with host resistance and still others that it develops because of secondary changes in the tumor such as necrosis and infection. In view of these differences of opinion and of the small number of studies that have been made, it seemed likely that a re-investigation of the matter might provide additional information that would help resolve some of the issues and so the present study of sinus cell hyperplasia was undertaken.

In the early years of the nineteenth century, a number of gynecologists noted that, even in the absence of metastases, enlargement of the pelvic lymph nodes sometimes accompanied carcinoma of the uterine cervix (Ries, 1901; Kroemer, 1904; Courtois-Suffit, 1901; Vinay, 1900; Schindler, 1906). These authors observed that the enlargement was sometimes, but not always, associated with "a septic, ulcerative process in the cancerous growth", and conversely that enlargement was not necessarily always present when there was ulceration of the cancer. Microscopic examination of such enlarged nodes disclosed sinus cell hyperplasia and large lymphoid follicles with conspicuous germinal centers. In addition Kroemer (1904) observed that the hyperplastic nodes remained free of cancer for long periods of time although sooner or later metastases developed. Because of this observation, he suggested that the reaction in the lymph node might act as a barrier to metastases, although he did not believe the evidence indicated that lymph nodes actually destroyed cancer cells. Kroemer's opinions

* Present address.

have received both pathological and experimental support (Goldmann, 1907 ; Yoffey, 1932). Schindler (1906) also reported that in his experience metastases were less common in lymph nodes that showed sinus cell hyperplasia than in those that did not. Vinay (1900) and Courtois-Suffit (1901), differentiated three forms of lymphadenopathy in cancer of the uterine cervix, 1) precancerous inflammatory enlargement and fibrosis, 2) cancerous lymphadenopathy, and 3) inflammatory lymphadenopathy. Most recent workers, however, have rejected the concept of a precancerous period of preparation of lymph nodes for metastases (Willis, 1934).

Despite the early recognition by gynecologists of alterations in the lymph nodes regional to a cancer, most students of tumors have paid little attention to the changes believing they were in no way distinctive and to be ascribed to a variety of causes : to the effects of microorganisms, to absorption of extravasated blood pigments or lipids, to retention of secretions, particularly in the breast (Ewing, 1940 ; Willis, 1934 ; Symmers, 1951 ; Marshall, 1956), to decomposition of the primary tumor (Kitain, 1922 ; Geller, 1950), to metabolic products of the primary tumor (Walther, 1948) or simply to " non-specific changes " (Willis, 1934).

In 1953, Black, Kerpe and Speer made a systematic study of the regional lymph nodes in 226 patients with mammary cancer and concluded that " the lymph nodes in an appreciable percentage of patients with breast cancer show marked sinusoidal and follicular histiocytic transformation as well as amyloid-like changes. Such changes are more prominent in cases with longer survival. A direct correlation was found also between the occurrence of such changes in the nodes and the survival of individual cases. On the other hand, no relationship was observed between the lymph node appearance and the age of the patient, or the histopathologic type of the primary tumor." They estimated that in approximately 20–40 per cent of cases of breast cancer, the axillary nodes exhibit sinus cell hyperplasia of moderate or marked degree. Among 19 patients who lived less than three years after operation, 79 per cent had no significant hyperplasia whereas among 53 patients who lived five years or more, 59 per cent had sinus cell hyperplasia. Black, Kerpe and Speer suggested these changes afford an accurate basis for prediction of five-year survival and that patients with marked sinus histiocytosis might be assured of a five-year cure in almost every case regardless of axillary metastases. These authors, however, noted that, even when the axillary nodes showed no sinus hyperplasia, 30 per cent of their patients lived at least five years after operation.

Berg (1956) confirmed the findings of Black, Kerpe and Speer with regard to lymph nodes regional to breast carcinoma, but interpreted the findings differently. Berg found that " when the size of the primary cancer and the amount of axillary metastases were held constant the degree of histiocytosis was the same whether or not the patients died of carcinoma within three years after radical mastectomy. It thus appears that the observed association of low histiocytosis and poor prognosis is indirect only and dependent upon known prognostic factors, primarily the quantity of axillary metastases and the time of operation." Berg also reported wide variations in the amount of histiocytosis in nodes from a given patient.

This survey of the literature makes it clear that an appreciable percentage of lymph nodes regional to a primary carcinoma may become enlarged even in the absence of metastases and that such nodes may show dilatation of lymphatic sinuses and hyperplasia of the cells lining them. The evidence also indicates that lymph nodes with sinus cell hyperplasis at the time of operation have fewer metastatic deposits than do nodes without hyperplasia and that statistically patients with sinus hyperplasia have a better prognosis than do patients without such hyperplasia. The meaning of these observations is, however, not agreed upon. Some observers hold that the sinus cell hyperplasia is an indication of host resistance while others doubt that the available evidence supports this idea.

In addition to sinus cell hyperplasia, some authors have also reported finding granulomatous lesions in the lymph nodes adjacent to a cancer (Wolbach, 1911; Nickerson, 1937; Nadel and Ackerman, 1950; Gherardi, 1950; Symmers, 1951; Black, Kerpe and Speer, 1953; ten Seldam, 1956; Wartman, 1956; Gorton and Linell, 1957). The granulomas consist of numerous epithelioid cells, a few multinucleated giant cells and small foci of fibrinoid necrosis. The lesions do not contain stainable micro-organisms and are found in regional but not in distant lymph nodes. The granulomas have been variously described as tuberculoid or sarcoid, but there is no evidence that they are due either to tubercle bacilli or are a part of generalized Broeck's sarcoidosis, and therefore the term "granuloma" seems preferable. The factors that cause the granulomas to form are unknown but the following have been considered: products of neoplastic growth or breakdown probably of lipid nature (Symmers, 1951; Refvern, 1954; ten Seldam, 1956; Gorton and Linnell, 1957), radiation of the primary tumor (Larsson, 1949; Gorton and Linnell, 1957), infection of the primary tumor and hypersensitivity (ten Seldam, 1956).

Deposits of hyaline or amyloid-like material in the sinuses and pulp of the lymph nodes have also been described and are thought to be in some way related to sinus cell hyperplasia or granuloma formation (Symmers, 1951; Black, Kerpe and Speer, 1953).

### MATERIAL AND METHODS

All cases of adenocarcinoma of the female breast recorded in the files of the Bland-Sutton Institute of Pathology, Middlesex Hospital, London, for the year 1951 were reviewed. The patients had been treated by radical mastectomy and in most cases histologic sections of the primary tumor and axillary lymph nodes stained with hematoxylin and eosin were available for microscopic study. In addition, follow-up studies had been made so that it was known whether the patients were (1) living and free of cancer, (2) living but with clinical evidences of cancer, (3) dead of cancer, or (4) dead of unrelated cause. Althogether there were 103 cases in the files and of these 66 had histologic sections of both the primary tumor and axillary lymph nodes (404 nodes) as well as adequate follow-up data. These 66 cases were analyzed with respect to (1) sinus cell hyperplasia in the lymph nodes, (2) metastases, (3) histologic grade of the primary tumor, (4) length of survival after operation, (5) inflammation and necrosis of the primary tumor, (6) follicular hyperplasia, (7) granuloma formation, and (8) hyaline deposits in the lymph nodes.

A similar analysis was made of patients with primary adenocarcinoma of the colon operated on at Middlesex Hospital during the year 1956. This particular year was chosen because a special study of carcinoma of the colon had been started that year and all lymph nodes removed by the surgeon had been prepared for microscopic examination. There were twenty-four cases with 303 lymph nodes.

The degree of sinus cell hyperplasia was graded empirically as absent, slight, moderate or marked, but for the purposes of the tables only nodes with moderate or marked changes were considered as showing sinus cell hyperplasia, whereas nodes with slight changes were classified with those showing none at all. Not every node in a given case showed sinus cell hyperplasia and at times the hyperplasis in individual nodes was focal rather than diffuse. Such variation, however, is not recorded in the tables which show simply whether or not hyperplasia was present in any of the nodes.

The primary carcinomas in both breast and colon were graded histologically according to the method of Patey and Scarff (1928, 1929) and Scarff (1952) which is based on the recognition of two factors—anaplasia and rapid growth. Anaplasia is recognized by absence of tubule formation ; and rapid growth by irregularity of nuclei, hyperchromatism and mitosis. By use of this method the tumors were sorted as follows : Grade 1 carcinomas showed only slight anaplasia and evidences of slow growth, Grade 2 carcinomas showed moderate anaplasia and evidences of moderately rapid growth and Grade 3 tumors anaplasia and rapid growth.

### RESULTS

Microscopic examination of the lymph nodes showed that 50 per cent of cases of breast carcinoma and 33 per cent of cases of colon carcinoma had sinus cell hyperplasia (Table I). The changes consisted of dilatation of the lymphatic sinuses, often most pronounced in the medullary sinuses, and hyperplasia, proliferation and desquamation of the cells lining the sinuses. These cells had abundant, faintly eosinophilic cytoplasm which often appeared amoeboid, stretching out and branching laterally at the poles of the cells. Some cells were continuous with similar ones on the wall of the sinus and formed a syncytium. The cells stained positively with the Weil-Davenport method, and were thought to be littoral cells (the metalophil cells of Marshall (1956) or the reticular cells of Maximow and Bloom (1942). These findings confirm the observations of others.

### *Correlation of Sinus Cell Hyperplasia with Metastases, Length of Survival and Histologic Grade of the Primary Tumor.*

Analysis of the cases according to whether or not tumor deposits were discovered in the nodes removed at operation showed that sinus cell hyperplasia occurred more frequently in patients free of metastases than in those with metastases (Table I). In breast carcinoma only 36 per cent of patients with sinus cell hyperplasia had metastases in contrast to 74 per cent of those without it. Similarly in colon carcinoma only 12 per cent of patients with sinus cell hyperplasia in the

TABLE I.—*General Incidence of Sinus Cell Hyperplasia and Correlation with Lymph Node Metastases and Survival*

| Lymph nodes | Per cent of total cases | Per cent of cases with positive nodes | Per cent of cases alive 7 years |
|---|---|---|---|
| | | *Breast Carcinoma* (66 *cases*) | |
| With hyperplasia | 50 | 36 | 64 |
| Without hyperplasia | 50 | 74 | 39 |
| | | *Colon Carcinoma* (24 *cases*) | |
| With hyperplasia | 33 | 12 | — |
| Without hyperplasia | 67 | 50 | — |

regional lymph nodes had metastases whereas 50 per cent of patients without sinus cell hyperplasia had metastases. It should be noted, however, that although the same relative differences were apparent, the absolute incidence was less in patients with colon carcinoma than in those with breast carcinoma.

Table I shows a similar difference with respect to survival in patients with breast carcinoma : 64 per cent of patients who had sinus cell hyperplasia at the time of operation were alive and apparently free of cancer seven years later, while only 39 per cent of those without sinus cell hyperplasis lived so long. Survival data for colon carcinoma were not available.

A positive correlation was also apparent for the histologic grade of the tumor and the occurrence of sinus cell hyperplasia for 64 per cent of Grade 1 carcinomas of the breast showed sinus cell hyperplasia but only 34 per cent of Grade 3 tumors (Table II). In carcinoma of the colon, 40 per cent of Grade 1 tumors showed sinus cell hyperplasis and none of Grade 3 tumors.

TABLE II.—*Correlation of Histologic Grade of the Primary Tumor with Sinus Cell Hyperplasia of Regional Lymph Nodes and Survival of Patients*

| | *Breast Carcinoma* | | | *Colon Carcinoma* | |
|---|---|---|---|---|---|
| | Per cent total cases | Per cent with hyperplasia | Per cent alive 7 years | Per cent total cases | Per cent total with hyperplasia |
| Total | — | 50 | 53 | — | 33 |
| Grade 1 | 21 | 64 | 57 | 42 | 40 |
| Grade 2 | 43 | 55 | 59 | 29 | 57 |
| Grade 3 | 35 | 34 | 43 | 29 | 0 |

*Correlation of Sinus Cell Hyperplasia with Inflammation and Necrosis of the Primary Tumor*

Repeatedly it has been suggested that inflammation and necrosis of the primary tumor cause sinus cell hyperplasia. The data bearing on this point from the present series of cases of breast and colon cancer have been set out in Tables III and IV.

TABLE III.—*Correlation of Inflammation or Necrosis of Primary Tumor with Sinus Cell Hyperplasia and Follicular Hyperplasia of Regional Lymph Nodes*

| | *Breast Carcinoma* | | | *Colon Carcinoma* | | |
|---|---|---|---|---|---|---|
| | Per cent total cases | Per cent with sinus hyperplasia | Per cent with follicular hyperplasia | Per cent total cases | Per cent with sinus hyperplasia | Per cent with follicular hyperplasia |
| With inflammation or necrosis | 37 | 56 | 13 | 71 | 29 | 27 |
| Without inflammation or necrosis | 63 | 50 | 20 | 29 | 43 | 35 |

Table III gives the percentage of cases with inflammation or necrosis of the primary tumor which also had hyperplasia. Although 37 per cent of breast carcinomas and 71 per cent of colon carcinomas showed either inflammation or necrosis, only a half of the breast and a quarter of the colon tumors had sinus cell hyperplasia in regional lymph nodes. Thus inflammation and necrosis were

TABLE IV.—*Correlation of Nodal Metastases with Follicular Hyperplasia, Granulomas and Hyaline Deposits*

| | Per cent total cases | Per cent with follicular hyperplasia | Per cent with granulomas | Per cent with hyaline |
|---|---|---|---|---|
| | | *Breast Carcinoma* | | |
| All cases | — | 4 | 5 | 26 |
| Cases with negative nodes | 44 | 6 | 9 | 36 |
| Cases with positive nodes | 56 | 2 | 2 | 17 |
| | | *Colon Carcinoma* | | |
| All cases | — | 29 | 8 | 3 |
| Cases with negative nodes | 62 | 37 | 13 | 7 |
| Cases with positive nodes | 38 | 33 | 0 | 0 |

not always associated with sinus cell hyperplasia and it would seem that other factors must have operated in these cases. Careful microscopic examination of the lymph nodes from the cases with inflamed or necrotic primary tumors also failed to reveal the expected evidences of inflammatory lymph adenopathy such as follicular hyperplasia (Table III), phagocytosis, lipid deposits, abscesses or accumulations of plasma cells.

*Follicular Hyperplasia, Granulomas and Hyaline*

A small proportion of cases of both breast and colon carcinoma showed in regional lymph nodes an increased number of large follicles with prominent germinal centers, granulomas of sarcoid type, or hyaline deposits (Table IV). Follicular hyperplasia occurred more frequently in carcinoma of the colon and hyaline deposition in carcinoma of the breast. In both types of cancer, granuloma and hyaline were more frequent in nodes that did not contain metastases than in those that did.

### DISCUSSION

In this study one half the patients with breast carcinoma and one third of those with colon carcinoma had sinus cell hyperplasia of regional lymph nodes at the time of operation and analysis of the cases indicated that patients with sinus cell hyperplasia had developed fewer metastases in regional lymph nodes than had patients without such changes. Further, in patients with both metastases and sinus cell hyperplasia, the hyperplasia occurred chiefly in the nodes in which no tumor was discovered. These findings confirm those of other workers (Black, Kerpe and Speer, 1953; Black and Speer, 1958; Berg, 1956). Comparison of the histologic grade of the primary tumor and the prevalence of sinus cell hyperplasia in the regional nodes showed that it was present in 64 per cent of Grade 1 tumours of the breast but in only 34 per cent of Grade 3 tumors. In colon carcinoma a similar difference was obvious for 40 per cent of Grade 1 tumors had sinus hyperplasia, but none of Grade 3 tumors. Patients with nodal sinus hyperplasia also had a better chance of surviving for seven years after operation than did patients without sinus cell hyperplasia.

Although these findings suggest that host resistance to the tumor may have occurred, we must not overlook the possibility that the changes in the lymph nodes represent a reaction not to the tumor but to something else. One possibility is that necrosis and inflammation in the primary tumor may have caused the reaction. This possibility was investigated but the data on it are not clear-cut perhaps because of the nature of the material which was studied. Thus only the written descriptions of the tumors and the ordinary microscopic sections were available for determining the presence and severity of the necrosis and inflammation and it is possible that they may have been overlooked in the gross examination of the specimens or that the histologic sections did not include the areas of inflammation and necrosis. Nevertheless some information on the point can be obtained by analysis of the cases in which inflammation and necrosis were discovered. For example, in breast cancer only about half the cases with necrosis and inflammation of the primary tumor showed sinus cell hyperplasia and in colon carcinoma somewhat less than a third (Table II). Necrosis and inflammation were commoner in colon than in breast carcinoma—71 as contrasted with 37 per cent—but the incidence of hyperplasia was just the reverse—29 per cent of colon and 56 per cent of breast carcinomas. The microscopic appearance of lymph nodes with sinus cell hyperplasia was also different from that commonly seen in inflammatory lymphadenopathy, since follicular hyperplasia was not frequent and there were no abscesses, phagocytes or collections of plasma cells. It might also be expected that carcinoma of the colon, being more frequently infected than carcinoma of the breast, would show more sinus cell hyperplasia in regional lymph nodes but this was not the case. These findings suggest that although necrosis and inflammation may be one cause of sinus cell hyperplasia they are probably not the only one.

Other possible causes of sinus cell hyperplasia, such as retention of secretions could not be studied in the present material. It might be pointed out, however, that although retention of secretions may be a possibility in breast carcinoma, it does not seem to be a likely cause in carcinoma of the colon, stomach or uterine cervix where sinus cell hyperplasia also occurs.

The significance of the findings of granulomas and deposits of hyaline in some of the lymph nodes is unclear. The data in Table V, which are too few to be statistically significant, suggest that granulomas and hyaline were more common in nodes showing sinus cell hyperplasia than in those without it as well as in nodes that were free of secondary carcinoma (Table V). There was, however, no marked

TABLE V.—*Correlation of Sinus Cell Hyperplasia with Occurrence of Follicular Hyperplasia, Granulomas and Hyaline in Regional Lymph Nodes*

| | *Breast* | | | *Colon* | | |
|---|---|---|---|---|---|---|
| | Per cent with follicular hyperplasia | Per cent with granulomas | Per cent with hyaline deposits | Per cent with follicular hyperplasia | Per cent with granulomas | Per cent with hyaline deposits |
| All cases | 5 | 8 | 35 | 29 | 8 | 4 |
| Sinus cell hyperplasia | 5 | 14 | 46 | 33 | 11 | 0 |
| No sinus cell hyperplasia | 5 | 3 | 22 | 27 | 7 | 7 |
| Negative nodes | 6 | 9 | 36 | 27 | 13 | 7 |
| Positive nodes | 2 | 2 | 17 | 33 | 0 | 0 |
| Inflammation and necrosis | 13 | 14 | 39 | 27 | 6 | 6 |
| No inflammation and necrosis | 20 | 4 | 33 | 35 | 14 | 0 |

increase in the number of granulomas formed or the amount of hyaline deposited when the primary tumor was necrotic or inflamed.

The results of this study, while agreeing in the main with those of Black and his colleagues, nevertheless differ in certain respects which may now be mentioned. Follicular hyperplasia did not occur as frequently in the cases in the present series as in Black's and there was a positive correlation between the histological grade of the primary carcinoma and the incidence of sinus cell hyperplasia. Black could not show such a correlation. The reasons for these differences in observations are not apparent but in the case of histologic grading, different methods of evaluation may have been used. Black and his colleagues suggested that the presence of sinus cell hyperplasia assured a patient of a five year survival. Our data for a seven year survival period do not show this although, in general, the presence of sinus cell hyperplasia seemed to indicate a favorable prognosis. The correlation, however, was not sufficiently good to permit prediction of life expectancy in individual cases. The data of Black and his colleagues also suggest that follicular hyperplasia, granuloma formation and hyaline deposition in regional lymph nodes are related to sinus cell hyperplasia. Our data, on the other hand, do not suggest such a relation.

### SUMMARY

The occurrence of hyperplasia of the cells lining the sinuses of lymph nodes regional to a primary carcinoma has been studied in 66 patients with breast carcinoma and 24 patients with colon carcinoma. The data have been analyzed with respect to metastases in the regional lymph nodes, the histologic grade of the primary tumor, the length of survival of the patients after operation and the amount of inflammation and necrosis in the primary tumor. The data appear to support the following conclusions :

1. Sinus cell hyperplasia of regional lymph nodes occurred in a considerable proportion of patients with carcinoma—a half of those with breast cancer and a third of those with colon cancer.
2. Patients without metastases in the nodes showed sinus cell hyperplasia twice as frequently as patients with metastases.
3. Sinus cell hyperplasia correlated with the histologic grade of the primary tumor—the higher the grade of malignancy the less the sinus cell hyperplasia.
4. These correlations applied to both breast and colon carcinoma, but were more striking in the former.
5. Sixty-four per cent of patients with breast carcinoma who had sinus cell hyperplasia were alive seven years after operation, whereas only 39 per cent of patients without sinus cell hyperplasia lived that long. A similar analysis of patients with colon cancer was not done because they had not yet been followed for more than a year.
6. Inflammation and necrosis of the primary tumor were found in 37 per cent of breast carcinomas and 71 per cent of colon carcinomas. The evidence, however, suggests that factors other than inflammation and necrosis may cause the sinus cell hyperplasia.
7. These findings together with similar observations reported by other workers suggest that sinus cell hyperplasia may be an indication of host resistance to a primary carcinoma rather than a non-specific alteration.

This work was done during tenure of a Leave of Absence at the Bland-Sutton Institute of Pathology, Middlesex Hospital, University of London. It is a pleasure to express my thanks to Northwestern University for generously making possible the Leave of Absence and to my hospitable hosts at the Bland-Sutton Institute. Mr. W. W. Richardson, of the Surgical Department of the Middlesex Hospital, helped with the histologic grading of the tumors and provided follow-up data about the patients.

### REFERENCES

Berg, J. W.—(1956) *Cancer*, **9**, 935.

Black, M. M., Kerpe, S. and Speer, F. D.—(1953) *Amer. J. Path.*, **29**, 505.

*Idem*, Opler, S. R. and Speer, F. D.—(1954) *Surg. Gynec. Obstet.*, **98**, 725.—(1955) *ibid.*, **100**, 543.—(1956) *Ibid.*, **102**, 599.

*Idem* and Speer, F. D.—(1957) ‘ Human Cancer.’ Chicago (The Year Book Publishers), p. 73.—(1958) *Surg. Gynec. Obstet.*, **106**, 163.

Courtois-Suffit, M. (1901) *Bull. méd., Paris*, **15**, 445.

Ewing, J.—(1940) ‘ Neoplastic Diseases ’. Philadelphia. (Saunders).

Fahr, T.—(1923) *Virchows Arch.*, **247**, 66.

Geller, F. C.—(1950) *Arch. Geschwulstforch.*, **2**, 171.

Gellhorn, G.—(1902) *Amer. Gynec.*, **1**, 476.

Gherardi, G. J.—(1950) *Arch. Path.*, **49**, 163.

Gnirs, L.—(1954) *Z. Krebsforsch.*, **60**, 94.

Goldmann, E.—(1907) *Proc. Roy. Soc. Med.*, **1**, 14, Surg. Sect.

Gorton, G. and Linell, F.—(1957) *Acta radiol., Stockh.*, **47**, 381.

Homburger, F.—(1948) *Science*, **107**, 648.

Kitain, H.—(1922) *Virchows Arch.*, **238**, 289.

Kroemer, P.—(1904) *Arch. Gynaek.*, **73**, 57.

Larsson, L. G.—(1949) *Acta radiol., Stockh.*, **31**, 17.

Marshall, A. H. E.—(1956) ‘ An Outline of the Cytology and Pathology of the Reticular Tissue ’. London (Oliver and Boyd), p. 120.

Maximow, A. A. and Bloom, W.—(1942) ‘ A Textbook of Histology ’. Philadelphia (W. B. Saunders Co.), p. 271.

Nadel, E. M. and Ackerman, L. V.—(1950) *Amer. J. clin. Path.*, **20**, 952.

Nickerson, D. A.—(1937) *Arch. Path.*, **24**, 19.

Patey, D. H. and Scarff, R. W.—(1928) *Lancet*, i, 801.—(1929) *Ibid.*, ii, p. 492.

Refvern, O.—(1954) *Acta med. scand.*, Suppl., 294.

Ries, E.—(1901) *Amer. J. Obstet., Dis. Wom.*, **44**, 29.

Robb-Smith, A. H. T.—(1946) *Spec. Rep. Ser. med. Res. Coun. Lond.*, No. 259.

Scarff, R. W.—(1952) *Arch. Middx Hosp.*, **2**, 174.

Schindler, R.—(1906) *Mschr. Geburtsh. Gynäk*, **23**, 502.

ten Seldam, R. E. J.—(1956) *Med. J. Aust.*, **1**, 916.

Symmers, W. St. C.—(1951) *Amer. J. Path.*, **27**, 493.

Vinay, C.—(1900) *Lyon. méd.*, **95**, 289.

Walther, H. E.—(1948) ‘ Krebsmetastasen ’. Basel (Benno Schwabe & Co.), p. 86.

Wartman, W. B.—(1956) ‘ Year Book of Pathology and Clinical Pathology ’. Chicago (Year Book Publishers), p. 74.

Willis, R. A.—(1934) ‘ The Spread of Tumors in the Human Body ’. London (J. & A. Churchill), p. 31.

Welbach, S. B.—(1911) *J. med. Res.*, **24**, 253.

Yoffey, J. M.—(1932) *Brit. med. J.*, ii, 1052.